# Characterization of *Aeromonas hydrophila* isolated from freshwater fish with control trial

**DOI:** 10.1038/s41598-025-19907-6

**Published:** 2025-09-23

**Authors:** Safaa M. Shabana, M. A. Rashed, Ayman A. Atia, Hala A. M. Abd El-Hady

**Affiliations:** 1 Bacteriology Unit, Kafrelsheikh Provincial Lab, Animal Health Research Institute (AHRI), Agricultural Research Center (ARC), Giza, Egypt; 2 Fish Diseases Unit, Kafrelsheikh Provincial Lab, Animal Health Research Institute, Agricultural Research Center (ARC), Giza, Egypt; 3 Pathology Unit, Kafrelsheikh Provincial Lab, Animal Health Research Institute, Agricultural Research Center (ARC), Giza, Egypt

**Keywords:** Aeromonas hydrophila, Freshwater fish, Antimicrobial susceptibility test, Virulence genes, Experimental challenge, Histopathology, Microbiology, Pathogenesis

## Abstract

*Aeromonas hydrophila* is an opportunistic pathogen that is highly important for freshwater fish. In the present study, two freshwater fish species Nile tilapia (*Oreochromis niloticus*) and Mullet (*Mugil cephalus*) collected from various fish farms in Kafrelsheikh Governorate, Egypt. The fish samples were examined to determine *Aeromonas hydrophila* presence (*A. hydrophila*). In addition, a treatment trial was conducted involving four groups of Nile tilapia fish, which treated with florfenicol (FFC) and oxytetracycline (OTC) based on the antimicrobial susceptibility test results. According to the findings, 12 (20%) *A. hydrophila* strains were isolated from a total of 60 collected fish samples (30 of Nile tilapia and Mullet with percentages of 30% and 10%, respectively). Based on species-specific 16 S rRNA genes, six (6) isolates were identified as *A. hydrophila* and carried aerolysin (*aer*A) and hemolysin (*hyl*A) virulence genes, with percentages of 83.3% and 50%, respectively. Whereas, the antimicrobial resistance gene results were *bla*TEM with percent (100%) and *aadA*1 (83.3%). Histopathological changes were significantly reduced in all assessed organs (liver, spleen, kidney, and gills) in the FFC group compared to the OTC-treated group. The prevalence of virulent and multidrug-resistant *A. hydrophila* in aquaculture poses significant risks to fish health, economic productivity, and public health.

## Introduction

Aquaculture was one of the most important food sources and provided animal-based nutrition to everyone and lessen the food insecurity caused by population expansion. Fish constituted approximately 16% of the animal protein sources consumed worldwide^1^. In particular, Nile tilapia and mugil fish aquaculture were the most significant food-delivering resources in Egypt, and Egyptian aquaculture was essential to the nation’s food supply^2^.

Bacterial diseases affecting aquatic organisms were considered one of the serious causes of delayed growth or mortality, which leads to economic losses^3^. One of the most common organisms in freshwater and saltwater environments was Aeromonas species^4^. Aeromonas species were opportunistic pathogens, often acting as secondary invaders in immunocompromised or diseased fish^5^. *Aeromonas hydrophila* (*A. hydrophila)* had been recognized as one of the riskiest microorganisms that could kill a range of fish varieties grown in aquaculture^6^and it could transmit the aeromoniasis zoonotic disease to humans who ingested infected fish and water^7^. Fish were a crucial factor in the transmission of Aeromonas to humans^8^.

*A. hydrophila* had been related to common clinical symptoms such as septicemia, extensive hemorrhages, body ulceration, tail and fin rot, and abdominal swelling^4^.

Also, Aeromonas species could infect humans through ingestion or ulceration, though human infections were uncommon. Clinical indicators of the disease in people include edema or swelling at the site of infection^9^and some clinical signs as muscular necrosis, cellulitis, and septicemia^10^. In humans, Aeromonas could cause diarrhea, lung infections, gastroenteritis, sepsis, and bacteremia.

The encoded virulence genes and toxins of Aeromonas species (e.g., aerolysin and hemolysin) were directly linked to the pathogenesis mechanism, as it allows the organism to attack, multiply, and then colonize the tissues of the host, thus causing the disease’s development^11^. Therefore, the detection of these virulence genes had been become necessary to determine the Aeromonas presence and their pathogenicity^12^.

To reduce the prevalence of disease in fish farming, several antimicrobial drugs were employed as growth promoters and preventative measures^13^. Accordingly, possible antibiotic abuse or overuse resulted in the emergence of bacteria resistant to antibiotics, a decline in the effectiveness of antibiotic treatment for diseases in humans and animals, the dissemination of resistance characteristics throughout the bacteria population, and the growth of several fish pathogens resistant to antibiotics across the globe^14^.

Aeromonas species could produce various *β*-lactamases for conferring resistance to *β*-lactams, which have been known as chromosomally mediated *β*-lactamases. In the case of class-A *β*-lactamases, the previous studies have shown different prevalence for genes encoding TEM-type *β*-lactamases in Aeromonas species according to isolation sources^15^.

Also, Aeromonas species carried numerous aminoglycoside resistance genes^16^these genes as *aad*A1, *aad*A2, and *sat*1 genes were encoded resistance to streptomycin, spectinomycin, and streptothricin, respectively. Each of *aad*A1 and *aad*A2 genes were investigated earlier in wastewater bacteria on mobile genetic elements (MGE)^17,18^.

Consequently, multidrug-resistant Aeromonas isolates pose a public health risk, as these isolates may be transmitted to humans through the consumption of contaminated food or through close contact with aquatic environments^19^.

Pathological findings of *A. hydrophila* have recently been investigated in certain fish kinds^20^.The liver, kidney, spleen, stomach, and gills of fish infected with *A. hydrophila* frequently exhibit progressive histopathological changes^21^. *A. hydrophila* caused hemocytes to aggregate with cell necrosis in the gills; hemocytes and pyknotic nuclei to aggregate heavily in the hepatopancreas; and the infected fish’s digestive tract to accumulate hemocytes at lower rates^22^.

Therefore, the objectives of this study were to investigate the prevalence of *A. hydrophila* in Nile tilapia (*Oreochromis niloticus*) and Mullet (*Mugil cephalus*) collected from fish farms in Kafrelsheikh Governorate, Egypt; detect virulence (*aerA*, *hlyA*) and antimicrobial resistance (*blaTEM*, *aadA1*) genes; and evaluate the therapeutic efficacy of florfenicol and oxytetracycline through histopathological and clinical evaluation following experimental infection in Nile tilapia fish.

## Materials and methods

### Samples collection

Just about 60 pooled samples from freshwater fish, 30 of each Nile tilapia (*Oreochromis niloticus*) and Mullet (*Mugil cephalus*), were gathered from naturally infected fish from farms in Kafrelsheikh Governorate, Egypt within period from June to November 2024. The fish weight of Nile tilapia and Mullet for samples was (50 ± 5 g) and (110 ± 2 g), respectively. The culture system type in fish farmers was monoculture as the two fish species were collected from the different farmers. The fish showed loss of scales, exophthalmia, tail erosions, skin darkness and skin hemorrhagic lesion, with appearance of ascites and enlarged in internal organs. The samples were delivered right away to the lab for bacteriological analysis in a refrigerated container after being sealed in sterile flexible bags.

### Isolation and phenotypic identification of *A. hydrophila*

Samples from the fish under examination’s liver, heart, kidney, gills, and spleen were collected as pooled samples aseptically and inoculated in trypticase soya broth (TSB) at 37 °C for 24 h aerobically. A loopful of the inoculated broth was streaked on Aeromonas agar then incubated at 37 °C for 24 h aerobically^23^and the pale green with darker cores and diameters ranging from 0.5 to 3.0 mm colonies were purified, and then morphological characters as well as shape, size, Gram staining, and motility of the isolates and biochemical identification tests including; catalase, oxidase, urease, indole, citrate utilization, H_2_S production, Voges–Proskauer test, and sugars fermentation were determined according to Macfaddin^24^.

### Antimicrobial susceptibility test

Each *A. hydrophila* isolate was tested against various antibacterial discs of amoxicillin (AMX), 25 µg; cefotaxime (CTX), 30 µg; streptomycin (S), 10 µg; oxytetracycline (T), 30 µg; trimethoprim-sulfamethoxazole (SXT), 25 µg; nalidixic acid (NA), 10 µg; ciprofloxacin (CIP), 5 µg; and florfenicol (FFC), 30 µg, which were used in the performed test (Oxoid). Bacterial suspension was prepared according to previous study^25^and its turbidity was visually compared to the 0.5 MacFarland requirements. According to Clinical Laboratory Standards Institute (CLSI) protocol^26^quantify the resistance, moderate, and susceptible patterns.

### Analysis of *A. hydrophila*’s molecular genetics

It was carried out at Dokki’s Animal Health Research Institute. The QIAamp DNA Mini Kit (Qiagen, Germany, GmbH) was used for DNA extraction following the guidelines of the manufacturer. The primers were provided by Metabion (Germany) (Table 1).

**Table 1:** Target gene primer sequences and cycle settings.

Target genes	Primers sequences	Amplified segment (bp)	Primary denaturation	Amplification (35 cycles)			Final extension	References
				Secondary denaturation	Annealing	Extension		
*A. hydrophila 16 S rRNA*	GAAAGGTTGATGCCTAATACGTA	685	94 °C 5 min	94 °C 30 s	50 °C 40 s	72 °C 45 s	72 °C 10 min	Gordon et al.^27^
	CGTGCTGGCAACAAAGGACAG							
*aer*A	CACAGCCAATATGTCGGTGAAG	326	94 °C 5 min	94 °C 30 s	52 °C 40 s	72 °C 40 s	72 °C 10 min	Singh et al.^28^
	GTCACCTTCTCGCTCAGGC							
*hyl*A	GGCCGGTGGCCCGAAGATACGGG	592	94 °C 5 min	94 °C 30 s	55 °C 40 s	72 °C 45 s	72 °C 10 min	Rozi et al.^29^
	GGCGGCGCCGGACGAGACGGGG							
*bla TEM*	ATCAGCAATAAACCAGC	516	94 °C 5 min	94 °C 30 s	54 °C 40 s	72 °C 45 s	72 °C 10 min	Colom et al. ^30^
	CCCCGAAGAACGTTTTC							
*aad*A1	TATCAGAGGTAGTTGGCGTCAT	484	94 °C 5 min	94 °C 30 s	54 °C 40 s	72 °C 45 s	72 °C 10 min	Randall et al.^31^
	GTTCCATAGCGTTAAGGTTTCATT							

Primers were utilized in a 25- µl reaction containing 12.5 µl of EmeraldAmp Max PCR Master Mix (Takara, Japan), 1 µl of each primer of 20 pmol concentration, 5.5 µl of water, and 5 µl of DNA template. The reaction was performed in an Applied biosystem 2720 thermal cycler. The uniplex PCR was used for the PCR amplification. The PCR procedures were carried out according to their references (Table 1). The PCR products were separated through gel electrophoresis with 1.5% agarose gel (Applichem GmbH, Germany). Using a gel documentation system (Alpha Innotech Biometra) for the gel showing, then the data was assessed through computer software.

### Experimental challenge

A total of 120 healthy Nile tilapia fish, weighing an average of 50 ± 3 g, were acquired from a nearby fish farm in the Kafrelsheikh Governorate, Egypt. Fish were divided into 4 equal groups (each group of 30 fish was kept in glass aquariums) and adapted for 2 weeks before the experiment. Fish were preserved in glass aquaria supplemented by dechlorinated water, the debris and wastes were siphoned daily, and about 50% of the aquarium water was changed with dechlorinated water day after day. Throughout the experimental trial, the fish were fed a commercial fish meal twice per day with a rate of 2% of their body weight (B.W.) ^32^. According to results extracted of the virulence genes and antimicrobial susceptibility test to drugs most commonly used in fish farms, both the injected *A. hydrophila* isolate and the two utilized antibiotics in the experiment were carefully chosen. Artificially, fish (90 fish out of 120 only) were infected with previously isolated virulent *A. hydrophila* from Nile tilapia fish samples, and an intraperitoneal injection of *A. hydrophila* cultured broth for 24 h (0.5 ml of 1 × 10^7^ CFU per fish) was used to induce the experimental infection^22^.

As previously mentioned, based on the results of antimicrobial susceptibility test, each of the florfenicol (FFC) (florfenicol 300 mg/ml, UCCMA Co., Egypt) and oxytetracycline (OTC) (oxytetracycline hydrochloride 5%, El Nasr Chem. Co., Egypt) utilized for therapy experiments in the lab.

The 1st group of fish served as negative control and was injected intraperitoneally with 0.5 ml of sterile phosphate buffered saline (PBS) (non-infected also non-treated group) (G1), the 2nd group was the positive control (infected but non-treated group) (G2), the 3rd group was infected and also treated with FFC (25 mg/kg BW) (G3)^33^ in diet, while the 4th group was infected and treated with OTC (83 mg/kg BW) (G4)^34^. Each dose of (1.25) ml of FFC was mixed with approximately 5 ml of vegetable oil and applied to the fish feed, while (25) ml of OTC was mixed with 5 ml of vegetable oil and added to the fish feed then left to dry. On the third day of the *A. hydrophila* experimental infection, the fish were fed FFC and OTC in food (in the form of floating pellets) shortly after the onset of clinical signs. The fish were fed both dietary antibiotics separately for 10 consecutive days.

All groups were monitored, and clinical symptoms and mortality were noted for 10 days after starting treatment. The mortality ratio (MR%) of each group was documented until the experiment was completed. MR% = (Dead fish number/Total population number) x 100. Fish samples were taken for histopathological investigation, and both surviving and dead fish were submitted for bacteriological and clinical analysis, and then samples were prepared for histopathological examination.

### Histopathological examination

Liver, spleen, kidney, and gills tissue samples from experimentally infected Nile tilapia fish (5 fish from each group) were collected and fixed in a 10% formalin buffered solution for 24 h., then displaced to 70% ethanol till preparation. The tissues were dried using a graded ethanol series and xylene. Then, the samples were sectioned (5 sections from each tissue sample), fixed in paraffin, stained with hematoxylin and eosin (H&E), and then inspected microscopically^35^.

## Results

### Bacteriological isolation and phenotypic identification of *A. hydrophila*

According to the Table 2 findings, 20% (12/60) of *A. hydrophila* was isolated from the pooled fish samples; 9 isolates were isolated from Nile tilapia (30% [9/30]), and 3 were isolated from Mullet *(*10% [3/30]). These results based on based on their morphological and biochemical characteristics. Microscopically, the bacteria appeared as Gram-negative, motile, straight rod-shaped bacilli. All isolates were biochemically similar except for citrate and lactose fermentation tests, where they were positive to catalase, oxidase, indole, Voges Proskauer, and fermented sucrose, glucose, maltose, and mannitol, whereas they were negative to urease, and H_2_S production.

**Table 2:** *A. hydrophila* incidence in nile tilapia and mullet.

Fish species	Total samples	Positive samples	Percentage of positive samples
Nile tilapia	30	9	30%
Mullet	30	3	10%
Total	60	12	20%

### Antimicrobial resistance patterns of *A. hydrophila*

The results in Table 3 showed that highly phenotypically resistance of *A. hydrophila* to amoxicillin (AMX) and cefotaxime (CTX) with a percentage of 75%, followed by streptomycin (S) and ciprofloxacin (CIP) with a percentage of 66.7%, and then nalidixic acid (NA) with a percentage of 50%. In contrast, 75% of the isolates were sensitive to florfenicol (FFC), followed by 58.3% sensitive to trimethoprim–sulfamethoxazole (SXT) and oxytetracycline (T) with a percentage of 41.6%.

**Table 3:** Phenotypic antimicrobial resistance of *A. Hydrophila.*

Antimicrobial drugs	A. hydrophila					
	Sensitive		Intermediate		Resistant	
	No.	%	No.	%	No.	%
Amoxicillin	2	16.7%	1	8.3%	9	75%
Cefotaxim	1	8.3%	2	16.7%	9	75%
Oxytetracycline	5	41.7%	4	33.3%	3	25%
Ciprofloxacin	4	33.3%	0	0%	8	66.7%
Nalidixic acid	3	25%	3	25%	6	50%
Streptomycin	1	8.3%	3	25%	8	66.7%
Trimethoprim-sulfamethoxazole	7	58.3%	4	33.3%	1	8.3%
Florfenicol	9	75%	3	25%	0	0%

The results in Table 4 showed that multidrug-resistant *A. hydrophila* (MDR) were isolated with a percent (75%) of amoxicillin, cefotaxime, streptomycin, and ciprofloxacin. Also, the MAR (multiple antibiotic resistance) index ranged from 0.25 to 0.75.

**Table 4:** Antimicrobial resistance patterns of *A. Hydrophila.*

*A. hydrophila* isolates	Resistance pattern								Resistance pattern	MAR Index*	MDR isolates**	
	AMX	CTX	T	CIP	NA	S	SXT	FFC			No.	(%)
1	R	I	S	S	S	R	S	S	AMX, S	0.25	–	(9 out of 12) (75%)
2	R	R	R	R	R	R	I	I	AMX, CTX, T, CIP, NA, S	0.75	1	
3	R	R	R	R	R	S	S	I	AMX, CTX, T, CIP, NA	0.62	1	
4	R	R	I	R	R	R	I	S	AMX, CTX, CIP, NA, S	0.62	1	
5	R	R	I	R	R	R	I	S	AMX, CTX, CIP, NA, S	0.62	1	
6	R	I	I	R	I	R	S	S	AMX, CIP, S	0.37	1	
7	I	R	R	R	R	R	I	S	CTX, T, CIP, NA, S	0.62	1	
8	R	R	S	R	I	I	S	S	AMX, CTX, CIP	0.37	1	
9	S	S	S	S	S	I	S	S	–	–	–	
10	S	R	S	S	S	R	S	S	CTX, S	0.25	–	
11	R	R	I	S	R	R	R	I	AMX, CTX, NA, S, SXT	0.62	1	
12	R	R	S	R	I	I	S	S	AMX, CTX, CIP	0.37	1	

*MAR Index (Multiple Antibiotic Resistance Index) = the number of antibiotics to which the isolates showed resistance/the total number of antibiotics screened.

**MDR: Multidrug resistance to at least three dissimilar antimicrobial classes.

### Identifying and utilizing polymerase chain reaction (PCR) to detect *A. hydrophila* virulence and antibiotic resistance genes

**Fig. 1:**
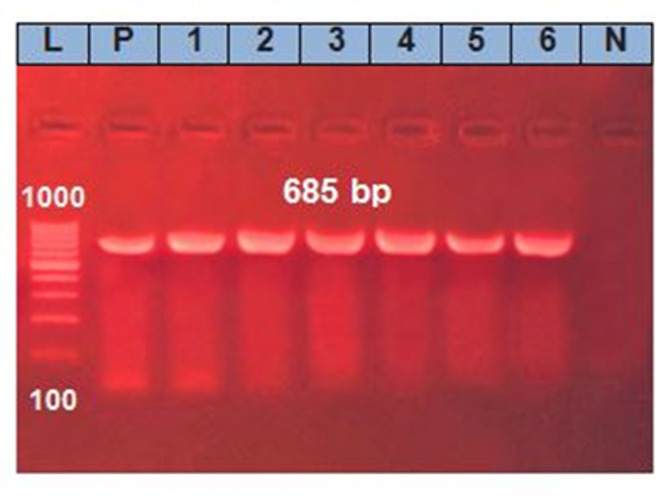
The polymerase chain reaction (PCR) result showed that lanes 1–6 were positive samples at 685 bp for the *A. hydrophila*-specific 16 S rRNA gene. Lane (L): 100 bp ladder; lanes (P), (N): control positive and negative.

Based on the molecular identification, six (6) isolates were identified using PCR for the species-specific 16 S rRNA gene of *A. hydrophila* with an amplicon size of 685 bp after biochemical confirmation. The results exhibited that all tested isolates (100%) were *A. hydrophila* organisms (Table 5; Fig. 1).

**Table 5:** Distribution of species-specific 16 S rRNA, virulence genes, and antibiotic resistance-encoding genes of *A. hydrophila* isolates.

Isolates	No. of isolates	Specific 16s rRNA gene		Virulence genes				Antibiotic resistance encoding genes			
				*aer*A		*hly*A		*bla*TEM		*aad*A1	
		No.	%	No.	%	No.	%	No.	%	No.	%
*A.hydrophila*	6	6 (6/6)	100	5 (5/6)	83.3	3 (3/6)	50	6 (6/6)	100	5 (5/6)	83.3

**Fig. 2:**
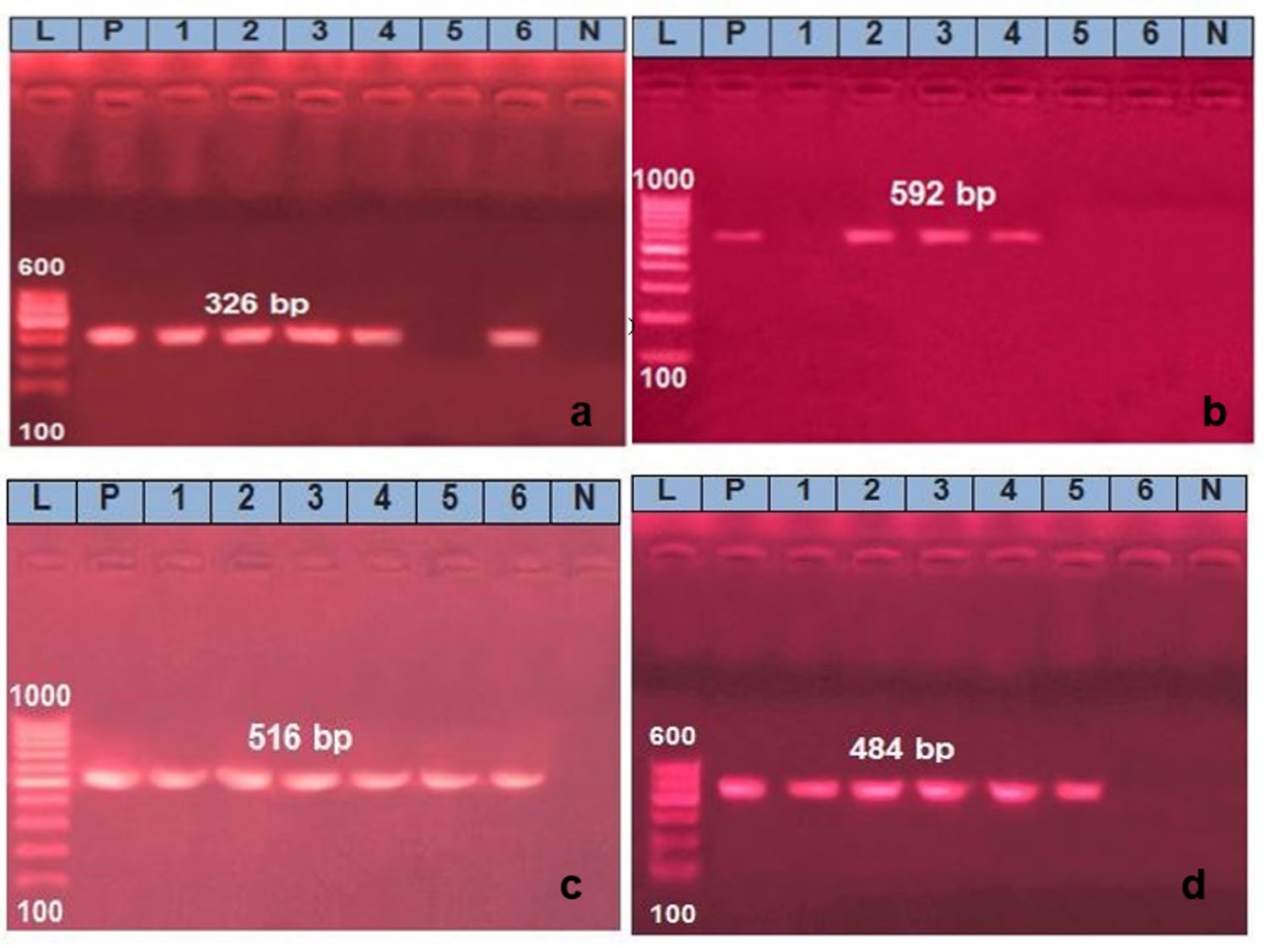
PCR with amplification of virulence and antibiotic-encoding resistance genes of *A. hydrophila* revealed (**a**), positive amplicons of *aer* A gene at lanes 1, 2, 3, 4, 6 at 326 bp but lane 5: negative samples (**b**), *hly*A gene positive amplicons at lanes 2, 3, 4 at 592 bp but lanes 1, 5, 6 negative samples (**c**), *bla*_TEM_ gene positive amplicons at lanes 1 to 6 at 516 bp and (**d**), *aad*A1 gene positive amplicons at lanes 1, 2, 3, 4, 5 at 484 bp but lane 6 negative samples. Lane (L) 100 bp ladder lanes (P), (N) control positive and negative.

The virulence genes, *aer*A (extracellular aerolysin A) and *hly*A (extracellular hemolysin A), were detected in the six *A. hydrophila* isolates that were identified using the PCR technique at bands 326 bp and 592 bp, respectively. The virulence gene results showed that *aer*A and *hly*A were detected with a percentage of 83.3% and 50%, respectively (Table 5; Fig. 2).

In contrast, the antibiotic resistance-encoding genes, the *bla*TEM gene and *aad*A1 gene, were noticed at bands 516 bp and 484 bp, respectively. The antibiotic resistance-encoding gene results in the 6 identified *A. hydrophila* isolates showing that, the *bla*TEM and *aad*A1 resistance genes were detected with a percentage of 100% and 83.3%, respectively, (Table 5; Fig. 2).

### The experimentally induced *A. hydrophila* infection and the post-treatment rate of mortality

**Fig. 3:**
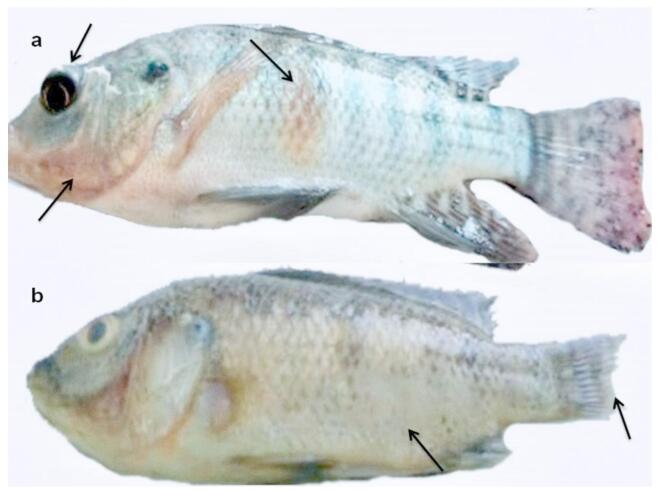
Nile tilapia fish infected with *A. hydrophila* (**a**), skin hemorrhagic lesion, hemorrhages in the opercular region and exophthalmia (**b**), loss of scales and tail erosions.

After infection with *A*. *hydrophila*, the Nile tilapia fish showed clinical symptoms such as erratic movement, loss of scales, exophthalmia, tail erosions, redness of skin, and hemorrhage in the opercular region. (Fig. 3).

**Fig. 4:**
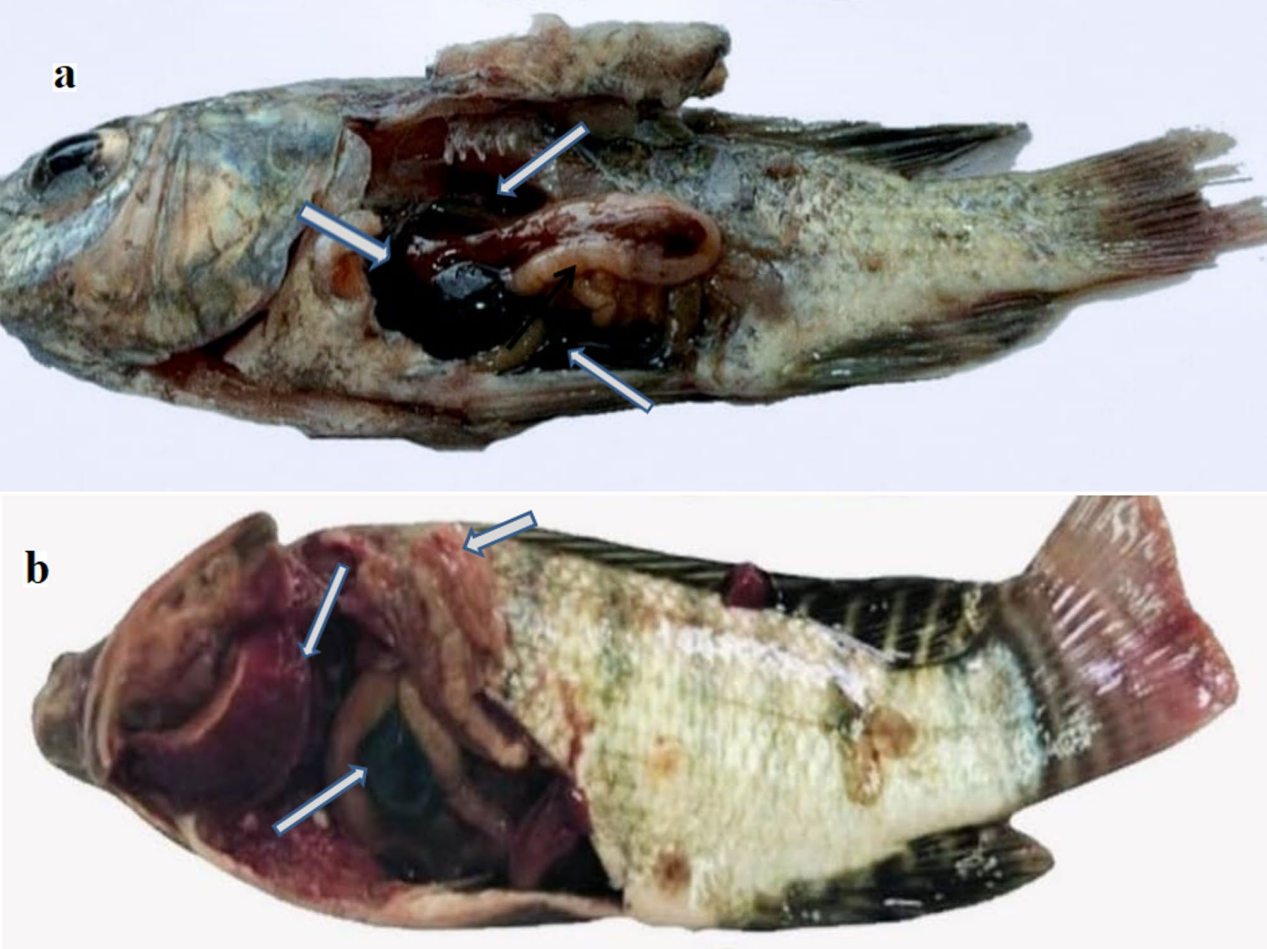
Post-mortem of Nile tilapia fish infected with *A. hydrophila* (**a**), severe congestion in the abdominal area and enlargement of internal organs (**b**), septicemia in the intestine, congested necrotic gills, and distended gall bladder.

The dead fish showed severe congestion in the abdominal area and enlargement of internal organs with septicemia in the intestine, congested necrotic gills, and distended gall bladder (Fig. 4).

At the conclusion of the experiment, the infected florfenicol-treated group exhibited no mortality, whereas the oxytetracycline group experienced 10% mortality compared to 40% in the control group (Table 6).

**Table 6:** The mortality rate of *A. hydrophila* infected fish used in the experiment at the end of treatment.

Groups	Fish No.	Antibiotic doses mg/kg B.W (In fish ration)	Dead fish No.	Mortality % (MR%)
G1: Non-infected and non-treated (Control negative)	30	–	–	–
G2: Infected and without treatment (Control positive)	30	–	12	40%
G3: Infected with florfenicol treatment	30	25 mg/kg B. W	0	0%
G4: Infected with oxytetracycline treatment	30	83 mg/kg B. W	3	10%

### Histopathological changes of experimentally challenged fish with *A*. *hydrophila*

**Fig. 5:**
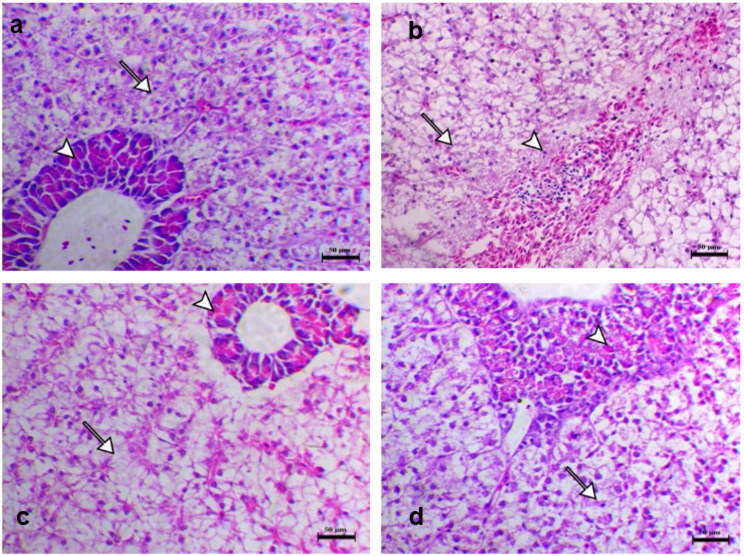
Liver histopathological sections of different groups (**a**), G1 showed the normality of hepatocytes and mild cytoplasmic vacuolation consisting of glycogen storage (arrow) and a normal pancreatic portion consisting of acinar pancreatic cells (arrowhead) (**b**), G2 showing a severe degree of hepatic vacuolation (arrow), blood congestion, and a complete necrotic pancreatic portion (arrowheads) (**c**), G3 showed decrease hepatic cells vacuolation (arrow), and mild atrophy of the pancreatic portion (arrowhead) (**d**), G4 showed decrease hepatic vacuolation (arrow) and mild to moderate granular degenerative changes within the pancreatic acinar cells (arrowhead). H&E stain, Bar = 50 μm.

**Fig. 6:**
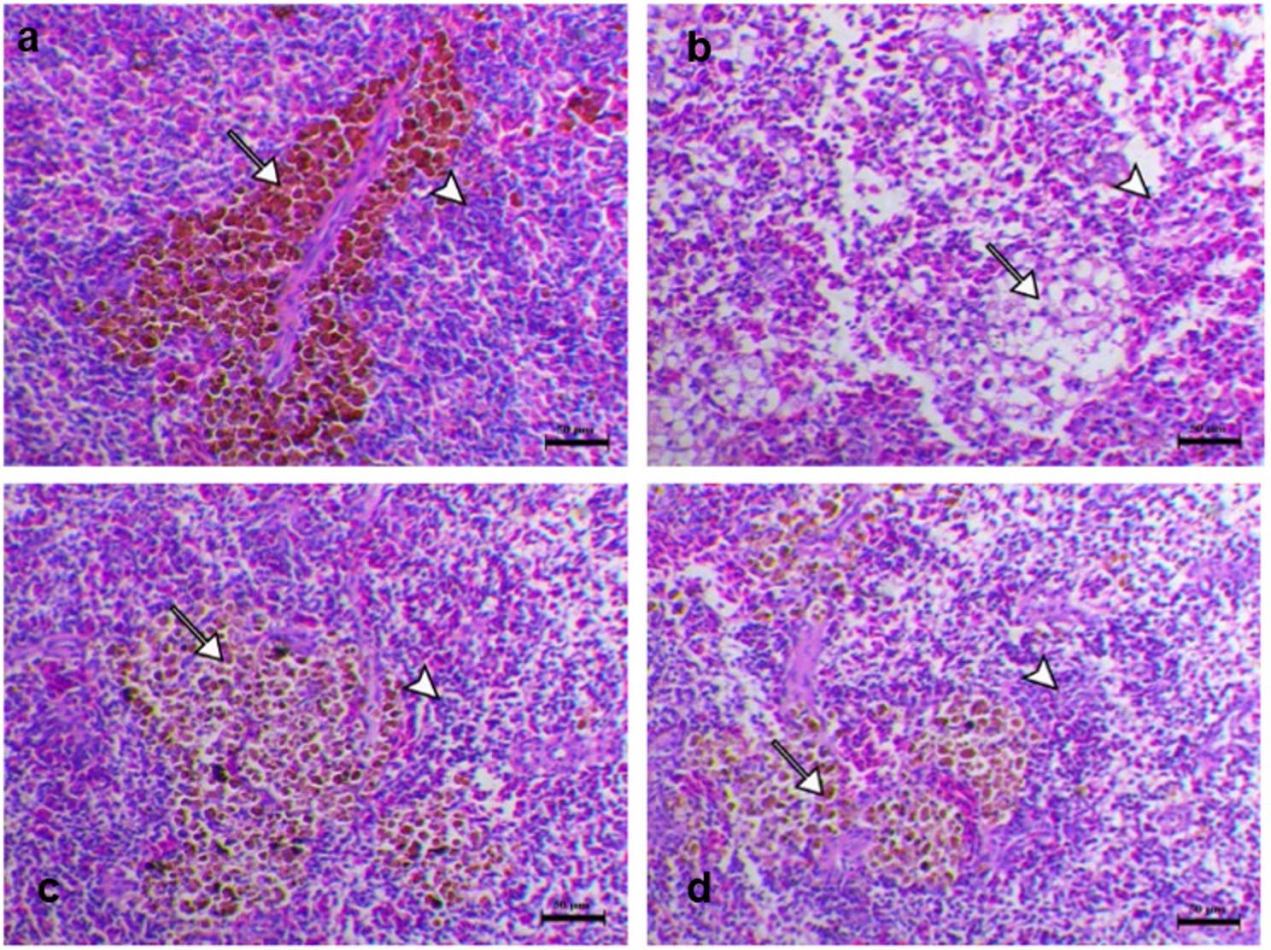
Spleen histopathological sections of different groups (**a**), G1 showed the normality of lymphoid components, lymphocytes (arrowhead), and normal melanomacrophage cells (arrow) (**b**), G2 showed severe necrotic lymphoid components associated with loss of both lymphocytes (arrowhead) and the complete disappearance of melanomacrophage cells (arrow) (**c**), G3 showed an intensification in the lymphoid cells (arrowhead) with a marked increase in melanomacrophage cells (arrow) (**d**), G4 showed an increase in the lymphoid cells (arrowhead) with a noticeable increase in the melanomacrophage cells (arrow). H&E stain, Bar = 50 μm.

**Fig. 7:**
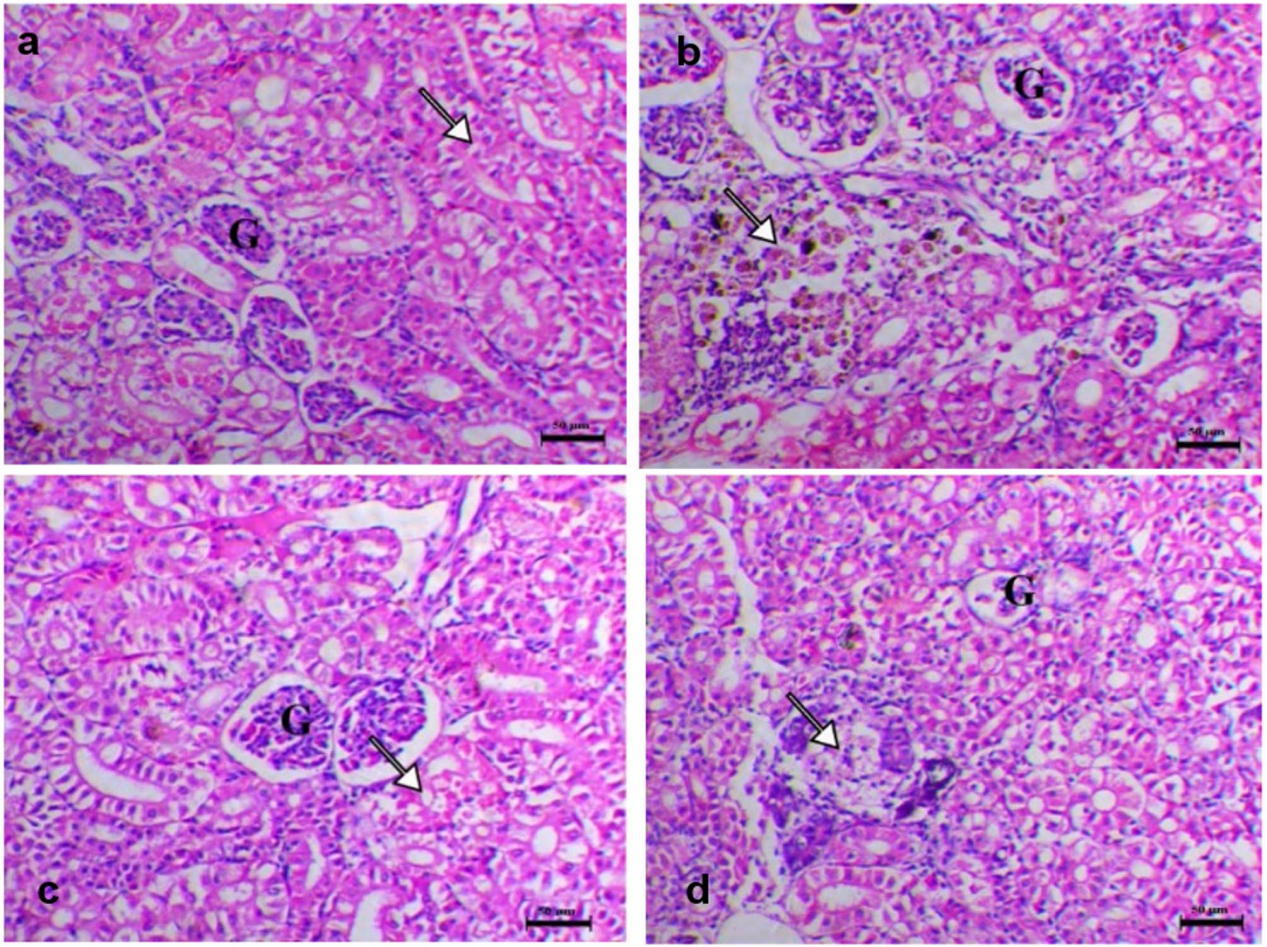
Kidney histopathological sections of different groups (**a**), G1 showed the normality of renal tubules (arrowhead) and glomeruli (G) (**b**), G2 showed interstitial nephritis associated with infiltration of melanomacrophage cells (arrow) (**c**), G3 showed focal eosinophilic degeneration within the renal tubules (arrow) and with normal glomeruli (G) (**d**), G4 showed focal renal tubular necrosis (arrow) and with mild glomerular atrophy (G). H&E stain, Bar = 50 μm.

**Fig. 8:**
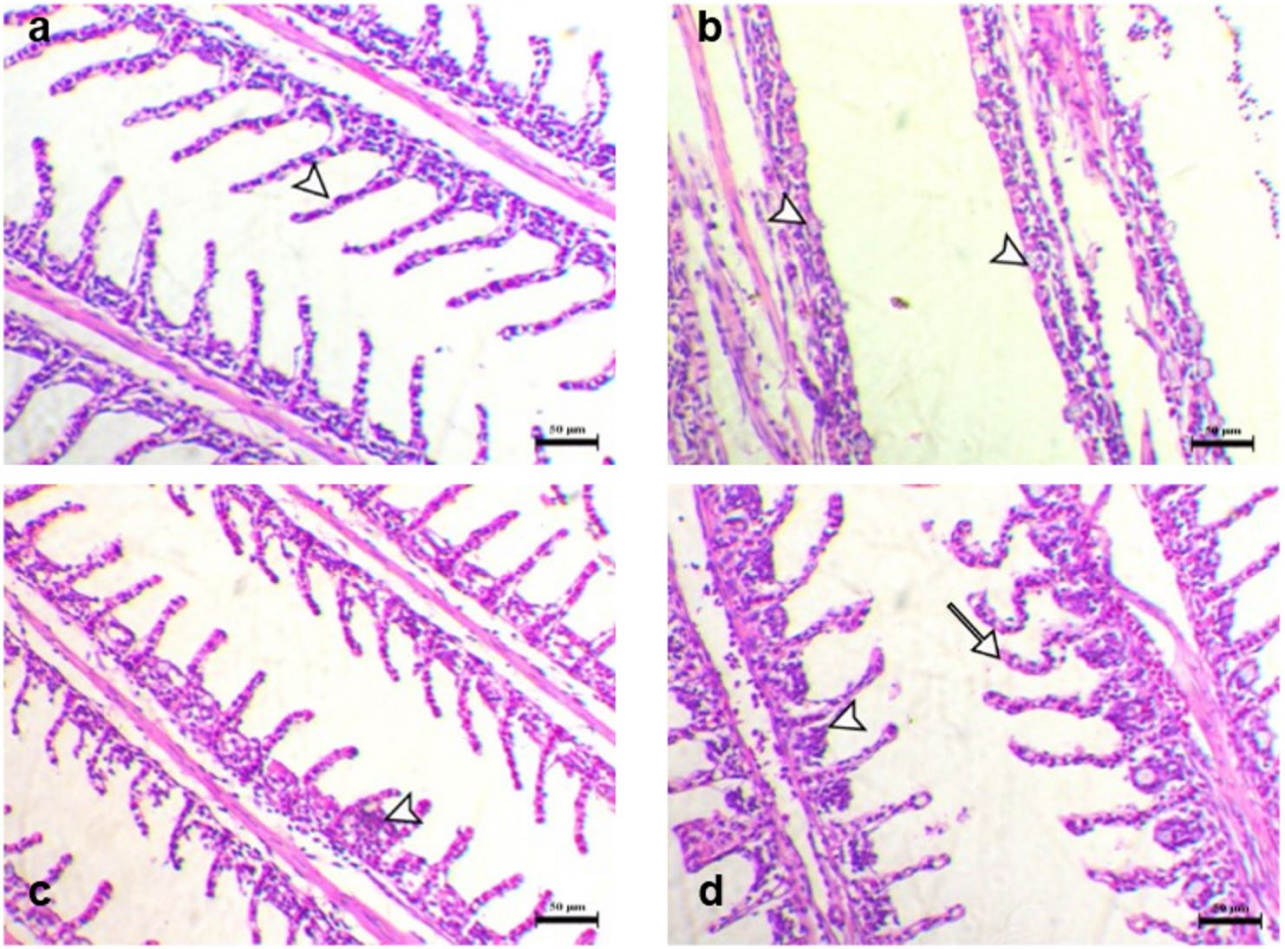
Gills histopathological sections of different groups (**a**), G1 showed the normality of secondary lamellae (arrowhead) (**b**), G2 showed noticeable loss, thickening, and the secondary lamellae adhesion with mucous cells proliferation (arrowheads) (**c**), G3 showed marked decrease with focal adhesion of the secondary lamellae (arrowhead) (**d**), G4 showed decrease of the secondary lamellae adhesion (arrowhead) with curving and bending of some lamellae (arrow). H&E stain, Bar = 50 μm.

Histopathological changes of the internal organs (liver, spleen, kidney, gills) were shown in Figs. 5, 6 and 7, and 8. Normal histological appearance of all the examined organs (liver, spleen, kidney, and gills) (Figs. 5a, 6a, 7a and 8a) was found in the control group (G1). In contrast, the livers of challenged fish with *A*. *hydrophila* (G2) showed several degrees of hepatic vacuolation, blood congestion, and complete necrosis of the pancreatic portion (Fig. 5b). The spleen of challenged fish showed several necroses of lymphoid components associated with loss of both lymphocytes and the complete disappearance of melanomacrophage cells (Fig. 6b). The kidney of challenged fish showed interstitial nephritis associated with infiltration of melanomacrophage cells (Fig. 7b). The challenged fish’s gills displayed a significant loss, thickening with adhesion of secondary lamellae and a proliferation of mucous cells (Fig. 8b). The experimentally challenged fish groups that were administered FFC and OTC (G3, G4) showed decreasing histopathological changes in all inspected organs (liver, spleen, kidney, gills) with a marked effect in the florfenicol treated group (Figs. 5c and d and 6 c, d, 7 c, d, and 8 c, d). As the result of the degeneration in tissue and blood vessels during the infection period, might appear severe necrotic lymphoid components in liver, spleen and kidney (Figs. 5c and d and 6 c, d, 7 c, d) and blood congestion in all inspected organs (liver, spleen, kidney, gills) (Figs. 5c and d and 6 c, d, 7 c, d, and 8 c, d) .

## Discussion

One of the most common pathogenic bacteria that infected fish was Aeromonas species, especially *A. hydrophila*, which has been linked to human disorders^2,36,37^.

Similar clinical symptoms and morphological changes were detected in moribund fish infected with *A. hydrophila* in the present study^38^. These findings also concurred with those of Ayoub et al. ^39^, who reported that a clinical symptoms of Nile tilapia fish naturally infected with Aeromonas spp. included extensive bleeding, including fin and tail tearing with body depigmentation and ocular opacity. Anemia, lethargy, anorexia, ulceration, and bleeding were some of the symptoms that might be linked to the small blood vessels rupturing caused by *A. hydrophila* infestation and the discharge of extracellular components.

All *A. hydrophila* isolates in this study showed typical morphological and biochemical characteristics of almost *A. hydrophila* that similar to those previously reported by Eid et al.^40^ and Abo-Shama et al. ^41^. However, *A. hydrophila* isolates showed dissimilar in the consumption of citrate and lactose that agree with Abo-Shama et al.^41^, this variation in biochemical characteristics might be due to the differences in the fish species and origin^42^and to the presence or absence of genes that regulate metabolic traits^43^.

The *A. hydrophila* incidence was 30% and 10% in Nile tilapia and Mullet fish respectively, in the present study, which agrees with the findings of El-Hawary et al.^44^ and Ayoub et al.^39^, who reported 32% and 15% prevalence percentage of *A. hydrophila* in Nile tilapia and Mullet fish, respectively, while the high prevalence of *A. hydrophila* was noted by Abdel-Moneam et al.^45^ at 81.3% in Nile tilapia and El-Hawary et al.^44^ at 38% in Mullet. Meanwhile, Ahmed et al.^46^ reported that *A. hydrophila* was isolated with a percent (6.25%) from Mullet and (4.67%) from Nile tilapia. The prevalence might vary depending on the fish types, the holding facilities, the sampling procedures, the region, and the management strategy.

The multi-antibiotic resistance observed in Aeromonas indicates an emerging health issue affecting both humans and aquatic animals^47^.

In the present study, most *A. hydrophila* isolates were resistant to amoxicillin (AMX) and cefotaxime (CTX) with a percentage (75%), which is similar to Elkamouny et al. ^48^,who noted that 100% of the *A. hydrophila* isolates under investigation were resistant to amoxicillin and 80% were resistant to ceftriaxone, and consistent with El-Hawary et al.^44^ and Samayanpaulraj et al.^49^, who noted that *A. hydrophila* was resistant to the *β*-lactam antibiotic class (ampicillin, penicillin G, methicillin, and amoxicillin), while differing from Sherif and Kassab^50^who noted that there were no isolates that were resistant to amoxicillin. Aeromonas species, a lactamase enzyme-producing species, might be linked to amoxicillin and cephalosporin resistance through the production of chromosomal lactamase^51^.

However, the isolated *A*. *hydrophila* were susceptible to florfenicol (FFC) with a percentage of 75%, followed by trimethoprim-sulfamethoxazole (SXT) with a percentage of 58.3%, which is similar to Sherif and Kassab^50^who noted that 83.3% of bacterial isolates were susceptible to florfenicol, and Saleh et al. ^52^, who noted that susceptibility was exhibited by trimethoprim-sulfamethoxazole with a percentage of 64.2%, while differing from El-Hawary et al.^44^, who reported no observed sensitivity to trimethoprim-sulfamethoxazole in all *A*. *hydrophila* isolates, and Elkamouny et al. ^48^, who reported that the isolated *A*. *hydrophila* were resistant to trimethoprim-sulfamethoxazole with a percentage of 65%.

In order to increase productivity, farmers constantly use a variety of antibiotic classes to prevent and manage harmful fish bacterial diseases^53^which might cause the development of bacteria strains resistant to various drugs.

Nine out of twelve isolates of *A. hydrophila* (75%) in the present study were resistant to three or more antimicrobial classes, making them multidrug-resistant isolates (MDR). This is consistent with Elkamouny et al. ^48^, who reported that 77.5% of multidrug-resistant *A. hydrophila* were isolated from seafood. However, higher (100%) prevalence of MDR *A. hydrophila* were previously reported^2^.

Because of the extensive use of antibiotics, in the present study, the MAR index values ranged from 0.25 to 0.75. This finding is consistent with that of Saleh et al. ^52^, who reported that the MAR index values ranged from 0.2 to 0.8 and El-Hawary et al.^44^, who reported that the MAR index of *A. hydrophila* > 0.2, while the index was 0.18 in the previous study of Sherif and Kassab^50^.

These results submitted that antibiotic treatment might soon lose its effectiveness due to widespread usage of the antimicrobial drugs to boost fish development in aquatic environments and lead to major consequences for public health.

The current PCR result of the specific 16 S rRNA gene of *A. hydrophila* revealed that all 6 isolates randomly selected (100%) were positive for *A. hydrophila*, parallel to the findings were reported by Abo Yadak et al. ^54^.

Virulence genes positive *A. hydrophila* strains constituted a serious public health risk since virulence factors associated with extracellular products were significant for the translocation in the epithelium^55^.

Aerolysin (*aerA)*, a pore-forming toxin, reduced the membrane permeability and caused osmotic lysis that results in cell death and had a significant role in the pathophysiology of *A. hydrophila*^56^. In addition, the hemolysin (*hly*A) was capable of lysing red blood cells and exhibited cytotoxic activity against a wide variety of species and cell types.

In regard to the detected virulent gene *aer*A, it was detected with a percent of 83.3% (5/6), while the *hly*A virulent gene was detected with a percent of 50% (3/6) in the 6 randomly tested *A. hydrophila* isolates in the current study, which comes in agreement with Nhinh et al.^57^, who detected the *aer*A gene with a percent of 83.9% in tilapia fish, and with Ramadan et al.^58^, who detected the *hly*A gene with a percent of 50%. On the other hand, this result is higher than that reported by El-Hawary et al.^44^, who noted that 62.5% of *A. hydrophila* isolates carried the *aer*A gene, and Ayoub et al.^39^ who reported that the *hly*A virulence gene with a percent of 7.9% of *A. hydrophila* isolates. In contrast, this result was lower than that reported by Hayati et al.^59^, who noted the *hly*A virulence gene in 95% of *A. hydrophila* isolates.

Although *β-*lactam antibiotics were typically the first choice for treating bacterial infections, their effectiveness has decreased over the past ten years as a result of bacterial strains producing mutations to be resistant to these antibiotics. The β-lactam resistance genes (*bla*TEM) were the most frequently detected *β*-lactam resistance genes.

Our study revealed that 100% and 83.3% of *A. hydrophila* isolates possessed *bla*TEM and aada1 genes (related to streptomycin resistance), respectively. These data were parallel with Eid et al.^40^, who reported the *bla*TEM gene in all isolates, while in another study of Ndi and Barton^60^*Aeromonas* strains from rainbow trout did not carry the *bla*TEM gene.

Similarly, the *aad*A1 resistance gene had been found at a higher rate of 70% ^60^ and 62.5% ^61^. However, Eid et al.^40^did not find the *aad*A1 gene in any isolated Aeromonas.

Therefore, the high rate of *β-lactamase* genes in the Aeromonas genus was the main cause of the high *β*-lactam antibiotic resistance in this genus^62^.

The broad-spectrum antibiotic FFC was used to treat a variety of bacterial illnesses in fish, and when administered as FFC in feed to Nile tilapia fish for 10 days, it would be well accepted Gaikowski et al. ^63^. OTC was one of the appropriate therapeutic agents for aquaculture usage. Chen et al. ^64^.

In the present study, the use of FFC and OTC with doses of 25 and 83 mg/kg BW/day administered in feed for 10 days, respectively, was effective in terminating experimental *A. hydrophila* infection in Nile tilapia. There were not any noted mortalities in the infected FFC-treated group (0%). In other research, Abu-Zahra et al. ^65^ reported that the mortality rate was 30% of Nile tilapia fish challenged with *A. hydrophila* that were administered FFC in fish meal for 10 days.

On the other hand, OTC treatment reduced the mortality in the present study to 10% compared with 40% in the control infected group, which agrees with Sherif et al. ^66^, who reported that the mortality rate reduction (MR%) of Nile tilapia experimentally infected with *A. hydrophila* and treated with OTC was 70% compared to 80% in controls.

In this study, the histopathological examination of the hepatopancreas of challenged fish with *A*. *hydrophila* (G2) showed several degrees of hepatic vacuolation, blood congestion, and complete necrosis of the pancreatic portion. The cause of hepatic cell vacuolation might be due to toxins produced by the causative pathogen^54^. The spleen of challenged fish showed several necroses of lymphoid components associated with loss of both lymphocytes and the complete disappearance of melanomacrophage cells, while the kidney showed interstitial nephritis associated with infiltration of melanomacrophage cells that agree with Yardimci and Aydin^67^who reported that congestion, degenerative changes, and inflammatory cell infiltration throughout the hepatic, splenic, and renal tissues occur as a pathological indication of acute septicemia induced by the *A. hydrophila* challenge.

Melanomacrophages were pigmented phagocytes originated primarily in the lymphoid tissues of poikilotherm as fish. Mostly accumulations of densely packed melanomacrophages, or melanomacrophage centers, were commonly aggregated in the kidney, spleen, and liver, while the splenic melanomacrophage centers were more commanded than kidney and liver one^68^and that is clearly shown in the histological appearance of the control group in our study.

In agreement with our results, Steinel and Bolnick^69^ reported that the aggregation of melanomacrophage cells in splenic and renal tissues is interrelated to the immune response of infected fish against the foreign microbial agents, where some virulent bacteria caused lymphoid cell necrosis including the melanomacrophage cells specific in early days of infection after that and with the treatment, they were beginning increase in number as the immune response of fish against the infection. Consequently, the appearance of melanomacrophage cells in control group and again disappeared in infected group is normally.

The gills of challenged fish showed a noticeable loss of thickening, with adhesion of the secondary lamellae and mucous cell proliferation that agrees with AlYahya et al. ^22^, who reported that infected gill lamellae showed hemocyte aggregation. According to another study by Azadbakht et al. ^70^, the main changes in the gills of *Acanthopagrus latus* exposed to different concentrations of *A. hydrophila* included, in certain situations, a thickening of the filament epithelium that filled the space between lamellae.

Histopathological changes in the examined organs (liver, spleen, kidney, and gills) decreased in the experimentally challenged fish group treated with FFC (G3). This finding matches with the results of Aboyadak et al. ^54^, who reported mild hepatic cell vacuolation without an inflammatory reaction, likely due to the drug’s antibacterial effect on the invading organism. In another study, Abu-Zahra et al. ^65^ revealed that the FFC-infected group showed mild hepatocyte vacuolation in addition to a significant increase in lymphoid with melanomacrophage cells in the splenic white pulp, while the renal tissues of Nile tilapia showed no change.

However, the histopathological changes of the hepatopancreas of the experimental challenged fish group treated with OTC showed decreased hepatic cell vacuolation with mild to moderate granular degenerative changes within the pancreatic acinar cells. Reda et al. ^71^ reported vacuolations in the hepatocytes and fatty changes of healthy Nile tilapia treated with OTC at a dose of 100 mg/kg ratio for 12 weeks. The oxytetracycline side effect and the effects of the infected pathogenic agent’s exotoxins might cause hepatic cell vacuolation.

## Conclusion

Therefore, it could be deduced that *Aeromonas hydrophila* is an important pathogen that causes septicemia in freshwater fish. Additionally, we perceived that the majority of *A. hydrophila* isolates from Kafrelsheikh Governorate, Egypt, included genes for both virulence and antibiotic resistance, and the production of these genes indicates its potential to cause adverse effects on fish production and public health. Both in vitro and in vivo investigations demonstrated that *A. hydrophila* was susceptible to FFC and OTC, with a marked effect of FFC. Finally, we recommended regular monitoring of the water and feed quality and accurate diagnosis and use of antibiotic drugs in the handling of fish infections to avoid the multiple antibiotic resistance (MAR) problem so that it controls the spreading of fish infections and mortality in Egyptian farms.

## Data Availability

The authors confirm that the data supporting the findings of this study are available within the manuscript, figures, and tables.
